# Evaluation of the Safety of Robot‐Assisted Radical Cystectomy for Bladder Cancer in Octogenarians

**DOI:** 10.1111/ases.70248

**Published:** 2026-01-30

**Authors:** Toshiharu Morikawa, Shuzo Hamamoto, Yoshihiko Tasaki, Daiki Ishikawa, Maria Aoki, Masakazu Gonda, Nobuhiko Shimizu, Takashi Nagai, Toshiki Etani, Taku Naiki, Ryosuke Ando, Kazuhiro Kanemoto, Atsushi Okada, Noriyasu Kawai, Tohru Mogami, Takahiro Yasui

**Affiliations:** ^1^ Department of Nephro‐Urology Nagoya City University Graduate School of Medical Sciences Nagoya Japan; ^2^ Department of Urology, Mie Prefectural Welfare of Agricultural Cooperatives Northern Mie Medical Center Komono Kousei Hospital Komono Japan; ^3^ Department of Clinical Pharmaceutics Nagoya City University Graduate School of Medical Sciences Nagoya Japan; ^4^ Department of Urology Nagoya City University West Medical Center Nagoya Japan; ^5^ Department of Urology Nagoya City University Midori Municipal Hospital Nagoya Japan

**Keywords:** aged, robotic surgical procedures, urinary bladder

## Abstract

**Introduction:**

Open radical cystectomy is the current standard treatment for bladder cancer. However, it is associated with high morbidity and mortality, particularly in the elderly. Recently, robotic surgery has become a minimally invasive approach. To this end, we aimed to evaluate the safety and complications of robot‐assisted radical cystectomy (RARC) in elderly patients with urothelial carcinoma.

**Methods:**

We performed a retrospective single‐center analysis of 103 patients who underwent RARC between May 2018 and May 2024. The patients were divided into an elderly group (age, ≥ 80 years; *n* = 24) and a younger group (*n* = 79).

**Results:**

The American Society of Anesthesiologists Physical Status Classification System scores were significantly lower in the elderly group than in the younger group. No significant differences were observed between the two groups in terms of demography. Operative time was shorter in the elderly group than in the younger group. Conversely, the postoperative hospital stay was shorter in the younger group than in the elderly group. There were no significant differences in the frequency or severity of complications between the two groups; however, the incidence of ileus was significantly higher in the elderly group. In addition, higher age, ileus, and days to drain removal were identified as independent factors that prolonged hospitalization.

**Conclusions:**

RARC is a safe treatment option for elderly patients with bladder cancer, with complication profiles comparable to those in younger patients. However, the increased risk of ileus and prolonged hospitalization in elderly patients highlights the need for cautious perioperative management to optimize outcomes in this growing population.

## 1 Introduction

Bladder cancer is the 10th most frequently diagnosed cancer worldwide and presents a substantial clinical challenge owing to its high recurrence rate. The current standard of care for muscle‐invasive bladder cancer and high‐risk non‐muscle‐invasive bladder cancer is radical cystectomy [1, 2, 3, 4]. Open radical cystectomy (ORC) is the conventional curative treatment in such cases. However, this procedure is often associated with high perioperative morbidity and mortality, particularly in elderly and/or frail patients [5, 6, 7, 8].

Recently, robotic surgery has become a minimally invasive approach in urology. Similarly, robot‐assisted radical cystectomy (RARC) was introduced to exceed ORC [9]. Although several reports showcase the safety and efficacy of RARC, its application in elderly patients remains controversial [10, 11]. In particular, several large‐scale studies have shown that ORC is reported to be associated with a significant risk of major complications and perioperative morbidity and mortality in patients aged ≥ 80 years [7, 8], even after adjustment for comorbidities and performance status. This age‐group also may require additional attention given evidence indiacting that octogenarians represent a clinically distinct population, with a nonlinear increase in mortality risk [12] and frailty prevalence [13] reflecting a marked decline in physiological reserves that directly impacts surgical tolerance and postoperative recovery. In an era of increasing life expectancy, a rapidly aging society, and a growing proportion of elderly people being diagnosed with bladder cancer, establishing the safety profile of RARC in this population is imperative for surgical decision‐making [12, 13, 14, 15]. Based on the above background, in this study, we aimed to evaluate the safety of RARC in patients aged ≥ 80 years.

## 2 Patients and Methods

### 2.1 Patients

A retrospective analysis was conducted in 103 patients presenting with urothelial carcinoma who underwent RARC at Nagoya City University Hospital between May 2018 and May 2024. The preoperative performance status was evaluated using the American Society of Anesthesiologists Physical Status Classification System (ASA‐PS) classification. Clinical staging was determined based on the pathological diagnosis of transurethral resection performed prior to radical cystectomy and imaging findings from enhanced computed tomography or magnetic resonance imaging. It was conducted in accordance with the TNM classification system, which is based on UICC version 8. Complications were graded according to the Clavien–Dindo classification system. The study protocol was approved by the Institutional Review Board of Nagoya City University Hospital (IRB approved number: 46‐17‐0007) and conformed to the provisions of the Declaration of Helsinki, 2024. Informed consent was obtained from all patients.

### 2.2 Statistical Analysis

Categorical variables were compared using Fisher's exact test, and quantitative variables were evaluated using the Mann–Whitney *U*‐test and Kruskal–Wallis test. Predictive factors for hospitalization for > 30 days were tested using logistic regression analysis. Multivariate logistic regression analysis was performed with *p* < 0.2 in the univariate logistic regression analysis. All tests were two‐sided, and *p* < 0.05 was considered statistically significant. All statistical analyses were performed using EZR software. This program is a graphical user interface for R [16].

## 3 Results

The 103 patients included in this study were divided into two groups for analysis: the Elderly group (*n* = 24), consisting of patients aged ≥ 80 years at the time of surgery, and the Younger group (*n* = 79), consisting of patients aged < 80 years. The demographic characteristics of the study population are shown in Table 1. The median age was 83.0 (81.0–84.0) years in the Elderly group and 73.0 (67.5–75.5) years in the Younger group. The ASA‐PS score was significantly lower in the Elderly group compared to the Younger group (*p* = 0.019). No significant differences were observed between the two groups in terms of body mass index, Barthel Index, clinical disease stage, preoperative chemotherapy status, regimen, or number of cycles administered. Furthermore, although the proportion of patients with comorbidities did not differ significantly between groups, the details of comorbidities were significantly more prevalent in the Elderly group.

**TABLE 1:** Patient characteristics.

	Overall (*n* = 103)	Elderly group (*n* = 24) (age ≥ 80)	Younger group (*n* = 79) (age < 80)	*p*
Age (years), median (IQR)	75.0 (69.0–79.0)	83.0 (81.0–84.0)	73.0 (67.5–75.5)	< 0.001*
Sex, *n* (%)				
Male	84 (81.6)	20 (83.3)	64 (81.0)	1.000
Female	19 (18.4)	4 (16.7)	15 (19.0)	
BMI, median (IQR)	23.6 (21.3–26.05)	24.4 (21.5–26.7)	23.6 (21.2–25.8)	0.376
ASA‐PS, *n* (%)				
1	3 (2.9)	3 (12.5)	0 (0.0)	0.019*
2	75 (72.8)	15 (62.5)	60 (75.9)	
3	25 (24.3)	6 (25.0)	19 (24.1)	
Barthel Index	100 (100–100)	100 (97.5–100)	100 (100–100)	0.768
Clinical T stage, *n* (%)^a^				
Carcinoma in situ	17 (16.5)	3 (12.5)	14 (17.7)	0.069
1 or lower	25 (24.3)	4 (16.7)	21 (26.6)	
2	55 (53.4)	12 (50.0)	43 (54.4)	
3	16 (15.5)	8 (33.3)	8 (10.1)	
4	7 (6.8)	0 (0.0)	7 (8.9)	
Clinical N stage, *n* (%)				
0	95 (92.2)	22 (91.7)	73 (92.4)	0.644
1	5 (4.9)	2 (8.3)	3 (3.8)	
2	3 (2.9)	0 (0.0)	3 (3.8)	
Neoadjuvant chemotherapy, *n* (%)				
Gemcitabine + cisplatin	63 (61.2)	17 (70.8)	46 (58.2)	0.598
Gemcitabine + carboplatin	7 (6.8)	1 (4.2)	6 (7.6)	
None	33 (32.0)	6 (25.0)	27 (34.2)	
Cycles of neoadjuvant chemotherapy, median (IQR)	3 (0–3)	3 (0.75–3)	3 (0–3)	0.094
Patients with comorbidities, *n* (%)^a^	84 (81.6)	21 (87.5)	63 (79.7)	0.551
Hypertension	42 (40.8)	11 (45.8)	31 (39.2)	0.012*
Hyperlipidemia	21 (20.4)	11 (45.8)	10 (12.7)	
Hyperuricemia	18 (17.5)	1 (4.2)	17 (21.5)	
Diabetes	26 (25.2)	10 (41.7)	16 (20.3)	
Heart disease	11 (10.7)	4 (16.7)	7 (8.9)	
Respiratory disease	6 (5.8)	1 (4.2)	5 (2.5)	
Hepatitis	3 (2.9)	1 (4.2)	2 (2.5)	
Stroke	7 (6.8)	1 (4.2)	6 (7.6)	
Other carcinoma	20 (19.4)	1 (4.2)	19 (24.1)	
Others	16 (15.5)	5 (20.8)	11 (13.9)	

Abbreviations: ASA‐PS, American Society of Anesthesiologists physical status classification system; BMI, body mass index; IQR, interquartile range.

^a^ Includes multiple stages.

* *p* < 0.05.

The perioperative factors are presented in Table 2. No significant differences were observed between the groups in terms of urinary diversion type, console time, days to oral intake, or days to pelvic drain removal. However, the operation time was shorter in the Elderly group (393.5 [343.5–449.8] min) compared to the Younger group (442.0 [405.5–522.5] min) (*p* = 0.0286). Regarding urinary diversion, the operative time for ureterocutaneostomy was significantly shorter in the Younger group. Conversely, the postoperative hospital stay was shorter in the Younger group (25.0 [21.5–31.5] days) compared to the Elderly group (34.5 [23.8–38.5] days) (*p* = 0.0318).

**TABLE 2:** Perioperative factors.

	Overall (*n* = 103)	Elderly group (*n* = 24) (age ≥ 80)	Younger group (*n* = 79) (age < 80)	*p*
Preoperative serum albumin	3.8 (3.6–4.2)	3.7 (3.5–4.1)	3.9 (3.6–4.2)	0.076
Preoperative serum CRP	0.130 (0.058–0.298)	0.130 (0.040–0.450)	0.100 (0.055–0.265)	0.931
Preoperative NLR	2.380 (1.492–3.166)	2.346 (1.578–2.771)	2.418 (1.492–3.482)	0.588
Urinary diversion, *n* (%)				
Ileal conduit (ICUD)	49 (47.6)	17 (70.8)	32 (40.5)	0.070
Ileal conduit (ECUD)	29 (28.2)	4 (16.7)	25 (31.6)	
Neobladder	7 (6.8)	0 (0.0)	7 (8.9)	
Cutaneous ureterostomy	18 (17.5)	3 (12.5)	15 (19.0)	
Operative time (min), median (IQR)	433.0 (381.5–512.5)	393.5 (343.5–449.8)	442.0 (405.5–522.5)	0.029*
Console time (min), median (IQR)	275.0 (221.0–365.0)	239.5 (224.0–334.0)	278.0 (220.0–365.5)	0.270
Days of resumption of oral intake, median (IQR)	3.0 (2.0–5.0)	3.0 (2.0–4.5)	3.0 (2.0–5.0)	0.416
Days of drain removal, median (IQR)	8.0 (7.0–14.0)	8.0 (7.0–15.25)	8.0 (7.0–14.0)	0.615
Days of hospitalization, median (IQR)	26.0 (22.0–36.5)	34.5 (23.8–38.5)	25.0 (21.5–31.5)	0.032*

Abbreviations: CRP, C‐reactive protein; ECUD, extracorporeal urinary diversion; ICUD, intracorporeal urinary diversion; IQR, interquartile range; NLR, neutrophil‐to‐lymphocyte ratio.

* *p* < 0.05.

Thirty‐day complications are shown in Table 3. There were no significant differences in the frequency and grade of complications between the two groups. However, the incidence of ileus was significantly higher in the Elderly group (*p* = 0.0180). Ileus was caused by the absence of intestinal peristalsis or motility in all cases; there were no cases of mechanical obstruction in this cohort.

**TABLE 3:** Complications.

	Overall (*n* = 103)	Elderly group (*n* = 24) (age ≥ 80)	Younger group (*n* = 79) (age < 80)	*p*
Overall complications, *n* (%)	67 (65.0)	16 (66.7)	51 (64.6)	1.000
Grade of complication (Clavien–Dindo)				
Grade 0	38 (36.9)	10 (41.7)	28 (35.4)	0.670
Grade I	20 (19.4)	6 (25.0)	14 (17.7)	
Grade II	31 (30.1)	6 (25.0)	25 (31.6)	
Grade III	14 (13.6)	2 (8.3)	12 (15.2)	
> Grade IV	0 (0.0)	0 (0.0)	0 (0.0)	
Type of complication				
Anemia	4 (3.9)	0 (0.0)	4 (5.1)	0.571
Fever	11 (10.7)	1 (4.2)	10 (12.7)	0.450
Wound infection	5 (4.9)	1 (4.2)	4 (5.1)	1.000
Pyelonephritis	10 (9.7)	2 (8.3)	8 (10.1)	1.000
Epididymitis	1 (1.0)	0 (0.0)	1 (1.3)	1.000
Pelvic inflammatory disease	4 (3.9)	1 (4.2)	3 (3.8)	1.000
Lymphorrhea	2 (1.9)	0 (0.0)	2 (2.5)	1.000
Ileus	14 (13.6)	7 (29.2)	7 (8.9)	0.018*
Ureteral obstruction	2 (1.9)	0 (0.0)	2 (2.5)	1.000
Anastomotic leakage	4 (3.9)	0 (0.0)	4 (5.1)	0.571
Parastomal hernia	2 (1.9)	1 (4.2)	1 (1.3)	0.413
Nausea	3 (2.9)	0 (0.0)	3 (3.8)	1.000
Decreased oxygenation	1 (1.0)	1 (4.2)	0 (0.0)	0.233
Deep vein thrombosis	1 (1.0)	0 (0.0)	1 (1.3)	1.000
Compartment syndrome	1 (1.0)	0 (0.0)	1 (1.3)	1.000

* *p* < 0.05.

**FIGURE 1:**
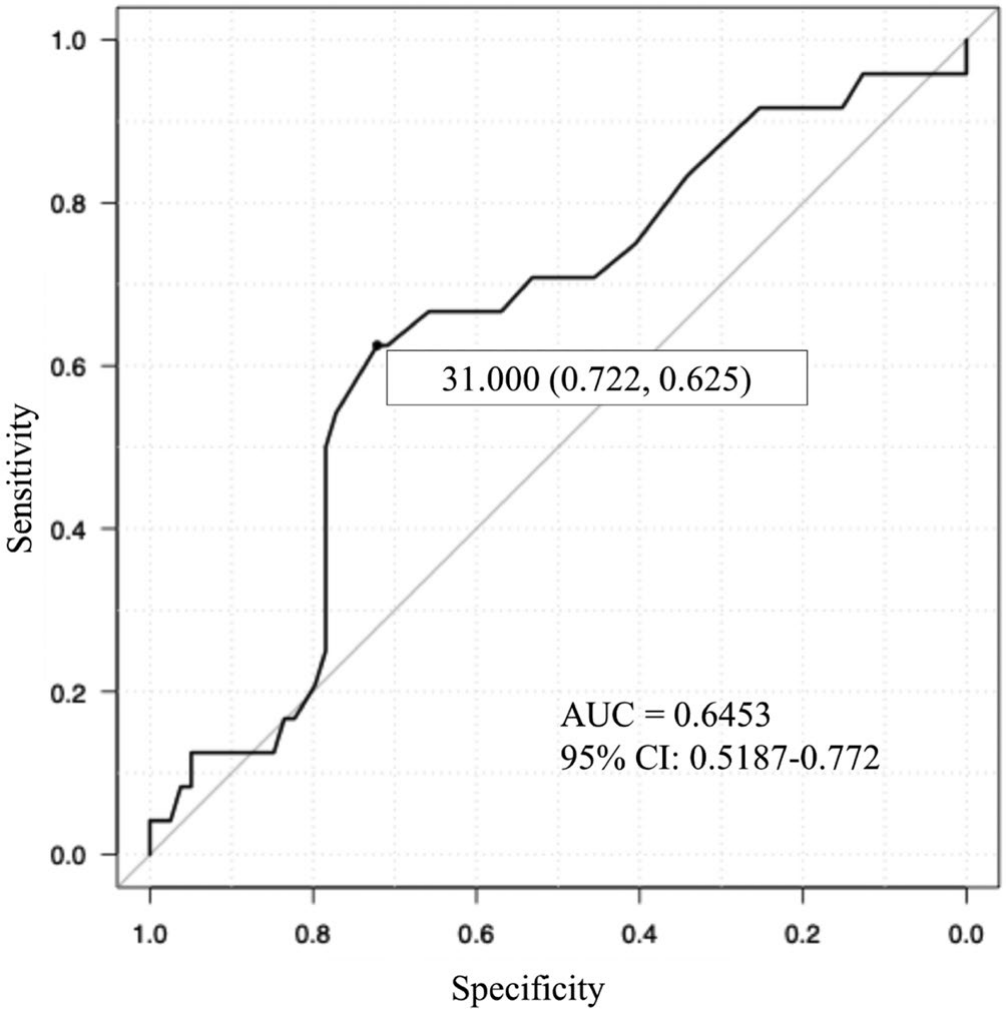
Setting cut‐off values using ROC curve analysis for days of hospitalization between the Elder and Younger groups.

The cutoff level for the duration of hospitalization was determined using a receiver operating characteristic curve targeting patients aged > 80 years (Figure 1). Univariate and multivariate analyses were conducted for each factor based on the identified cutoff of 31 days (Table 4). The analyses revealed that ileus, number of days to drain removal, and age were independent factors. However, neither operative time nor the type of urinary diversion was identified as an independent factor.

**TABLE 4:** Univariate and multivariate logistic regression analysis predicting the days of hospitalization over 30 days.

Factors	Univariate		Multivariate		
	Odds ratio (95% CI)	*p*	Odds ratio (95% CI)	*p*	VIF
Intercept			0.000123 (0.0000000538–0.279)	0.022*	
Age	1.070 (1.010–1.140)	0.017*	1.08 (1.01–1.16)	0.032*	1.178
Male sex	0.556 (0.203–1.520)	0.253			
BMI	1.040 (0.930–1.170)	0.467			
ASA‐PS class III or more	1.960 (0.782–4.900)	0.152	2.78 (0.877–8.820)	0.083	1.152
Preoperative serum Albumin	0.493 (0.204–1.200)	0.118	1.14 (0.368–3.540)	0.819	1.146
Preoperative serum CRP	1.050 (0.784–1.410)	0.737			
Preoperative NLR	0.936 (0.764–1.150)	0.520			
Urinary diversion					
Ileal conduit (ICUD)	2.110 (0.596–7.480)	0.246			
Ileal conduit (ECUD)	1.510 (0.462–4.930)	0.495			
Neobladder	0.433 (0.041–4.570)	0.486			
Cutaneous ureterostomy	1.000 (reference)	1.000 (reference)			
Operative time	0.997 (0.993–1.000)	0.255			
Complications (Clavien‐Dindo Grade 3 or more)	2.710 (0.915–8.020)	0.072	3.21 (0.772–13.3)	0.109	1.142
Ileus	8.880 (2.290–34.500)	0.002*	6.56 (1.03–41.80)	0.047*	1.485
Days of resumption of oral intake	1.110 (1.010–1.230)	0.028*	1.02 (0.89–1.18)	0.748	1.381
Days of drain removal	1.120 (1.050–1.200)	0.001*	1.12 (1.04–1.21)	0.004*	1.094

Abbreviations: ASA‐PS, American Society of Anesthesiologists physical status classification system; BMI, body mass index; CRP, C‐reactive protein; ECUD, extracorporeal urinary diversion; ICUD, intracorporeal urinary diversion; NLR, neutrophil‐to‐lymphocyte ratio.

* *p* < 0.05.

Moreover, univariate and multivariate analyses were performed to explore the predictive incidence of ileus (Table 5). Additionally, age was identified as a predictive factor for ileus.

**TABLE 5:** Univariate and multivariate logistic regression analysis predicting incidence of ileus.

Factors	Univariate		Multivariate		
	Odds ratio (95% CI)	*p*	Odds ratio (95% CI)	*p*	VIF
Intercept					
Age	1.170 (1.040–1.310)	0.007*	1.160 (1.030–1.300)	0.012*	1.002
BMI	0.939 (0.793–1.110)	0.466			
Male sex	0.804 (0.201–3.220)	0.757			
ASA‐PS class III or more	0.831 (0.212–3.250)	0.790			
Preoperative serum albumin	0.306 (0.092–1.020)	0.054*	0.369 (0.092–1.480)	0.159	1.002
Preoperative serum CRP	1.080 (0.746–1.570)	0.681			
Preoperative NLR	1.000 (0.787–1.280)	0.986			
Urinary diversion					
Ileal conduit (ICUD)	2.720 (0.279–26.500)	0.389			
Ileal conduit (ECUD)	3.320 (0.385–28.600)	0.275			
Neobladder	2.830 (0.152–52.700)	0.485			
Cutaneous ureterostomy	1.000 (reference)	1.000 (reference)			
Operative time	1.000 (0.994–1.010)	0.944			
Complications (Clavien–Dindo Grade 3 or more)	1.590 (0.391–6.500)	0.515			
Days of drain removal	1.030 (0.971–1.100)	0.304			

Abbreviations: ASA‐PS, American Society of Anesthesiologists physical status classification system; BMI, body mass index; CRP, C‐reactive protein; ECUD, extracorporeal urinary diversion; ICUD, intracorporeal urinary diversion; NLR, neutrophil‐to‐lymphocyte ratio.

* *p* < 0.05.

To further validate whether ileus risk increases progressively with age, we divided the patients into three subgroups based on a cutoff of 70 years—Group A: patients aged ≥ 80 years, Group B: patients aged between 70 and 80 years, and Group C: patients aged less than 70 years. Regarding patient characteristics, significant differences were observed only in age, ASA‐PS score, and details of comorbidities (Table S1). Furthermore, although no differences were observed in perioperative factors (Table S2) and overall incidence of complications, multiple comparisons revealed a significant difference in the incidence of ileus (*p* = 0.024) (Table S3).

## 4 Discussion

Our findings revealed that, even in elderly patients, RARC resulted in only a slight extension of hospital stay, with reduced operation time and without significantly affecting the incidence or severity of overall complications. Postoperative ileus was an independent risk factor for prolonged hospital stay. Importantly, this study was conducted in a patient cohort with significantly favorable ASA‐PS scores among the elderly population, and surgery was performed in elderly patients aged 80 years or older who were in good general condition. De Groote et al. [17] reported that patients aged ≥ 80 years undergoing RARC for bladder cancer did not have a higher risk of peri‐ and postoperative morbidity and mortality rate and had a similar 3‐year recurrence‐free survival. Xie et al. [18] also reported that elderly patients who underwent RARC had a similar risk of perioperative complications and recurrence‐free survival as younger patients. These studies suggest that RARC could be performed in elderly patients with complication rates and oncologic outcomes similar to those in younger patients.

In conventional ORC and laparoscopic radical cystectomy (LRC), no differences in overall or severe complications have been reported in elderly patients, as evidenced in our study [19, 20, 21]. However, in contrast to the RARC in our report, it has been noted that there is no difference in operative time between ORC and LRC, but it could be shorter in elderly patients undergoing ORC [20]. This may have been influenced by surgical strategies to reduce burden, such as choosing simple urinary diversion or reducing lymph node dissection in elderly patients [22]. A probability for the differences in the results of this study might be due to the effect of bias, as this study was conducted on a small sample size from a single institution.

Previous studies have reported inconsistent operative times. However, our study showed that the Younger group had longer operative times than the Older group. This might be because younger patients had higher clinical T and N stages and underwent more difficult surgeries, or because the proportion of neobladders in urinary diversion was higher and the surgical procedures were more complicated.

Prolonged hospitalization of elderly patients can lead to several challenges. Elderly patients are at an increased risk for loss of muscle strength and mobility due to minimal mobility caused by hospitalization, which might hamper their routine lifestyle even after discharge [23]. Furthermore, approximately 30% of elderly patients experience decreased functional independence in daily activities as a result of hospitalization [24]. On the mental aspect, there is a possibility that the delirium caused due to long‐term hospitalization may cause chronic deterioration of cognitive functions in elderly patients that might progress to dementia [25].

A novel aspect of this study is that it identified postoperative ileus and prolonged drain placement as potential factors contributing to an extended hospital stay. The study also found that early initiation of oral intake was effective in preventing ileus. Zennami et al. [26] also reported that early ambulation and oral intake after RARC may help prevent postoperative ileus. Additionally, enhanced recovery after surgery, initially developed as a multidisciplinary approach to promote postoperative recovery in gastrointestinal surgery, has been adapted to RARC and found to be advantageous in preventing ileus [27, 28]. Notably, among the factors contributing to a hospital stay exceeding a month, preoperative factors such as ASA‐PS and perioperative factors such as urinary diversion type were not identified as significant components. Instead, only age and postoperative factors were identified. These findings suggest that elderly patients may have ileus, longer hospital stays, and related challenges, highlighting the need for judiciously designed postoperative management.

This study had two limitations. First, this was a single‐center retrospective study. Consequently, there may be variations in the operative skills of the surgeons and assistants, which could introduce differences in surgical proficiency. Additionally, selection biases, such as variations in operative time due to patient factors and urinary diversion type, cannot be entirely ruled out. Secondly, the relatively small sample size may have limited the statistical power of the analysis.

Despite these limitations, our report of RARC in elderly patients provides valuable insights. The finding that overall complication rates were not significantly affected is crucial for designing treatment regimens. Furthermore, the identification of prolonged hospital stays and the need for ileus prevention highlights the importance of meticulous postoperative management in optimizing outcomes for elderly patients undergoing RARC. No differences were observed between groups even in terms of the preoperative Barthel Index, a comprehensive geriatric assessment index that focuses on physical function. The Barthel index has also been reported to be useful for evaluating postoperative complications in abdominal surgery [29], and our results are consistent with this finding. Furthermore, although there were no significant differences in comorbidities between the two groups in this cohort, the Elderly group showed significantly more detailed complications, with a higher proportion of lifestyle‐related diseases such as hypertension and hyperlipidemia. While heart disease, including acute coronary syndrome and arrhythmia, was more prevalent in the Elderly group, the ASA‐PS score indicated better outcomes in this group, suggesting no issues with activities of daily living. The lower proportion of other carcinoma in the elderly group tends to suggest selection bias due to patient factors.

In analyses further subdividing age groups, significant differences were observed only for age, ASA‐PS score, detail of comorbidities, and the occurrence of ileus. The tendency for ASA‐PS score to decrease with increasing age might represent a limitation of this study as a potential selection bias. Differences in the detail of comorbidities also did not significantly alter the overall trends between the two groups. The novel finding from these analyses further strongly indicates that increasing age is associated with an increased risk of ileus.

In conclusion, RARC is a safe treatment option for elderly patients with bladder cancer with complication profiles comparable to those in younger patients. However, the increased risk of ileus and prolonged hospitalization in elderly patients highlights the need for cautious perioperative management to optimize outcomes in this growing population.

## Author Contributions

**Toshiharu Morikawa:** methodology (equal), data curation (lead), formal analysis (lead), writing – original draft (lead). **Shuzo Hamamoto:** conceptualization (lead), methodology (lead), formal analysis (equal), writing – original draft (equal), writing – review and editing (lead). **Yoshihiko Tasaki:** writing – review and editing (equal). **Daiki Ishikawa:** writing – review and editing (equal). **Maria Aoki:** writing – review and editing (equal). **Masakazu Gonda:** writing – review and editing (equal). **Nobuhiko Shimizu:** writing – review and editing (equal). **Takashi Nagai:** writing – review and editing (equal). **Toshiki Etani:** writing – review and editing (equal). **Taku Naiki:** writing – review and editing (equal). **Ryosuke Ando:** writing – review and editing (equal). **Kazuhiro Kanemoto:** writing – review and editing (equal). **Atsushi Okada:** supervision (equal). **Noriyasu Kawai:** supervision (equal). **Tohru Mogami:** supervision (equal). **Takahiro Yasui:** supervision (lead).

## Funding

The authors have nothing to report.

## Ethics Statement

The study protocol was approved by the Institutional Review Board of Nagoya City University Hospital (IRB approved number: 46‐17‐0007) and conformed to the provisions of the Declaration of Helsinki, 2024. Informed consent was obtained from all patients.

## Consent

All patients provided informed consent.

## Conflicts of Interest

The authors declare no conflicts of interest.

## Supporting information


**Table S1:** Patient characteristics.
**Table S2:** Perioperative factors.
**Table S3:** Complications.

## Data Availability

The data that support the findings of this study are available from the corresponding author upon reasonable request.
