# Supplementary material for: Evaluation of the Safety of Robot‐Assisted Radical Cystectomy for Bladder Cancer in Octogenarians
Source: Asian J Endosc Surg. 2026 Jan 30;19(1):e70248. doi: 10.1111/ases.70248 (PMC12858331; doi:10.1111/ases.70248)
Supplement: Supplementary file 1 — Table S1: Patient characteristics. Table S2: Perioperative factors. Table S3: Complications. [file ASES-19-e70248-s001.docx]

**Supplementary Table 1. Patient characteristics**

|  | Group A  (Age ≥ 80)  (n = 24) | Group B  (70 ≤ age < 80)  (n = 51) | Group C  (Age < 70)  (n = 28) | P-value |
| --- | --- | --- | --- | --- |
| Age (years), median (IQR) | 83.0 (81.0-84.0) | 75.0 (73.0-77.0) | 65.0 (57.5-68.0) | <0.001* |
| Sex, n (%) |  |  |  |  |
| Male | 20 (83.3) | 39 (76.5) | 25 (89.3) | 0.382 |
| Female | 4 (16.7) | 12 (23.5) | 3 (10.7) |  |
| BMI, median (IQR) | 24.4 (21.5-26.7) | 23.3 (21.0-25.7) | 23.7 (21.5-26.1) | 0.674 |
| Barthel Index | 100 (100-100) | 100 (97.5-100) | 100 (100-100) | 0.952 |
| ASA-PS, n (%) |  |  |  |  |
| 1 | 3 (12.5) | 0 (0.0) | 0 (0.0) | 0.02* |
| 2 | 15 (62.5) | 42 (82.4) | 18 (64.3) |  |
| 3 | 6 (25.0) | 9 (17.6) | 10 (35.7) |  |
| Clinical T stage, n (%) † |  |  |  |  |
| Carcinoma in situ | 3 (12.5) | 6 (11.8) | 7 (25) | 0.221 |
| 1 or lower | 4 (16.7) | 13 (25.5) | 8 (28.6) |  |
| 2 | 12 (50.0) | 28 (54.9) | 15 (53.6) |  |
| 3 | 8 (33.3) | 6 (11.8) | 2 (7.1) |  |
| 4 | 0 (0.0) | 4 (7.8) | 3 (10.7) |  |
| Clinical N stage, n (%) |  |  |  |  |
| 0 | 22 (91.7) | 48 (94.1) | 25 (89.3) | 0.528 |
| 1 | 2 (8.3) | 1 (2.0) | 2 (7.1) |  |
| 2 | 0 (0.0) | 2 (3.9) | 1 (3.6) |  |
| Neoadjuvant chemotherapy, n (%) |  |  |  |  |
| Gemcitabine + Cisplatin | 17 (70.8) | 28 (54.9) | 18 (64.3) | 0.769 |
| Gemcitabine + Carboplatin | 1 (4.2) | 4 (7.8) | 2 (7.1) |  |
| None | 6 (25.0) | 19 (37.3) | 8 (28.6) |  |
| Cycles of neoadjuvant chemotherapy, median (IQR) | 3 (0.75-3) | 3 (0-3) | 3 (0-3) | 0.967 |
|  |  |  |  |  |
| Patients with comorbidities, n (%) † | 21 (87.5) | 42 (82.4) | 21 (75.0) | 0.536 |
| Hypertension | 11 (45.8) | 25 (49.0) | 9 (32.1) | 0.030* |
| Hyperlipidemia | 11 (45.8) | 7 (13.7) | 3 (10.7) |  |
| Hyperuricemia | 1 (4.2) | 12 (23.5) | 5 (17.9) |  |
| Diabetes | 10 (41.7) | 9 (17.6) | 7 (25.0) |  |
| Heart disease | 4 (16.7) | 6 (11.8) | 1 (3.6) |  |
| Respiratory disease | 1 (4.2) | 4 (7.8) | 1 (3.6) |  |
| Hepatitis | 1 (4.2) | 2 (3.9) | 0 (0.0) |  |
| Stroke | 1 (4.2) | 6 (11.8) | 0 (0.0) |  |
| Other carcinoma | 1 (4.2) | 12 (23.5) | 7 (25.0) |  |
| Others | 5 (20.8) | 5 (9.8) | 6 (21.4) |  |

†: includes multiple stages. *: p<0.05

BMI, body mass index; ASA-PS, American Society of Anesthesiologists physical status classification system; IQR: Interquartile range

**Supplementary Table 2. Perioperative factors**

|  | Group A  (Age ≥ 80)  (n = 24) | Group B  (70 ≤ age < 80)  (n = 51) | Group C  (Age < 70)  (n = 28) | P-value |
| --- | --- | --- | --- | --- |
| Preoperative serum albumin | 3.7 (3.5-4.1) | 3.8 (3.6-4.2) | 4.0 (3.8-4.3) | 0.072 |
| Preoperative serum CRP | 0.130 (0.040-0.450) | 0.150 (0.060-0.380) | 0.090 (0.045-0.163) | 0.424 |
| Preoperative NLR | 2.346 (1.578-2.771) | 2.486 (1.777-3.594) | 2.017 (1.253-2.811) | 0.448 |
| Urinary diversion, n (%) |  |  |  |  |
| Ileal conduit (ICUD) | 17 (70.8) | 19 (37.3) | 13 (46.4) | 0.138 |
| Ileal conduit (ECUD) | 4 (16.7) | 19 (37.3) | 6 (21.4) |  |
| Neobladder | 0 (0.0) | 4 (7.8) | 3 (10.7) |  |
| Cutaneous ureterostomy | 3 (12.5) | 9 (17.6) | 6 (21.4) |  |
| Operative time (min), median (IQR) | 393.5 (343.5-449.8) | 444.0 (401.5-510.0) | 432.5 (412.8-547.8) | 0.078 |
| Console time (min), median (IQR) | 239.5 (224.0-334.0) | 297.0 (225.0-418.0) | 240.5 (215.0-323.3) | 0.096 |
| Days of resumption of oral intake, median (IQR) | 3.0 (2.0-4.5) | 2.0 (2.0-4.5) | 3.0 (2.0-5.0) | 0.279 |
| Days of drain removal, median (IQR) | 8.0 (7.0-15.25) | 8.0 (7.0-15.00) | 8.0 (7.0-13.00) | 0.833 |
| Days of hospitalization, median (IQR) | 34.5 (23.8-38.5) | 25.0 (22.0-37.0) | 24.5 (18.8-29.0) | 0.051 |

*: p<0.05

CRP, C-reactive protein; NLR, neutrophil-to-lymphocyte ratio; ICUD, intracorporeal urinary diversion; ECUD, extracorporeal urinary diversion; min, minutes; IQR: interquartile range

**Supplementary Table 3. Complications**

|  | Group A  (Age ≥ 80)  (n = 24) | Group B  (70 ≤ age < 80)  (n = 51) | Group C  (Age < 70)  (n = 28) | P-value |
| --- | --- | --- | --- | --- |
| Overall complications, n (%) | 16 (66.7) | 33 (65.0) | 18 (64.3) | 1 |
| Grade of complication (Clavien–Dindo) |  |  |  |  |
| Grade 0 | 10 (41.7) | 18 (35.3) | 10 (35.7) | 0.545 |
| Grade I | 6 (25.0) | 10 (19.6) | 4 (14.3) |  |
| Grade II | 6 (25.0) | 18 (35.3) | 7 (25.0) |  |
| Grade III | 2 (8.3) | 5 (9.8) | 7 (25.0) |  |
| >Grade IV | 0 (0.0) | 0 (0.0) | 0 (0.0) |  |
| Type of complication |  |  |  | 0.255 |
| Anemia | 0 (0.0) | 4 (7.8) | 0 (0.0) | 0.122 |
| Fever | 1 (4.2) | 7 (13.7) | 3 (10.7) | 0.461 |
| Wound infection | 1 (4.2) | 2 (3.9) | 2 (7.1) | 0.902 |
| Pyelonephritis | 2 (8.3) | 6 (11.8) | 2 (7.1) | 0.778 |
| Epididymitis | 0 (0.0) | 1 (2.0) | 0 (0.0) | 0.601 |
| Pelvic inflammatory disease | 1 (4.2) | 2 (3.9) | 1 (3.6) | 0.994 |
| Lymphorrhea | 0 (0.0) | 1 (2.0) | 1 (3.6) | 0.651 |
| Ileus | 7 (29.2) | 6 (11.8) | 1 (3.6) | 0.024* |
| Ureteral obstruction | 0 (0.0) | 0 (0.0) | 2 (7.1) | 0.067 |
| Anastomotic leakage | 0 (0.0) | 1 (2.0) | 3 (10.7) | 0.085 |
| Parastomal hernia | 1 (4.2) | 1 (2.0) | 0 (0.0) | 0.558 |
| Nausea | 0 (0.0) | 2 (3.9) | 1 (3.6) | 0.626 |
| Decreased oxygenation | 1 (4.2) | 0 (0.0) | 0 (0.0) | 0.193 |
| Deep vein thrombosis | 0 (0.0) | 1 (2.0) | 0 (0.0) | 0.601 |
| Compartment syndrome | 0 (0.0) | 1 (2.0) | 0 (0.0) | 0.601 |

*: p<0.05
